# The Sequestration of Oxy-Polybrominated Diphenyl Ethers in the Nudibranchs *Miamira magnifica* and *Miamira miamirana*

**DOI:** 10.3390/md14110198

**Published:** 2016-10-27

**Authors:** Ariyanti S. Dewi, Karen L. Cheney, Holly H. Urquhart, Joanne T. Blanchfield, Mary J. Garson

**Affiliations:** 1 School of Chemistry and Molecular Biosciences, The University of Queensland, St. Lucia 4072, QLD, Australia; ariyanti.dewi@uqconnect.edu.au (A.S.D.); j.blanchfield@uq.edu.au (J.T.B.); 2 Research and Development Center for Marine and Fisheries Product Competitiveness and Biotechnology, Ministry of Marine Affairs and Fisheries, Jakarta 10260, Indonesia; 3 School of Biological Sciences, The University of Queensland, St. Lucia 4072, QLD, Australia; k.cheney@uq.edu.au (K.L.C.); holly.urquhart@uqconnect.edu.au (H.H.U.)

**Keywords:** oxy-polybrominated diphenyl ethers, nudibranch, Chromodorididae, *Miamira magnifica*, *Miamira miamirana*

## Abstract

A series of oxy-polybrominated diphenyl ethers (O-PBDEs) has been isolated from the extracts of *Miamira magnifica* and *Miamira miamirana* collected from Queensland, Australia. *M. magnifica* sequesters the new OH-PBDE **1** and six known OH-PBDEs containing four to six bromines (**2**–**7**). *M. miamirana* also accumulates known tribromo- and tetrabromo OMe-PBDEs **8**–**10** in both mantle and viscera tissues. To date, *Miamira* is the only genus of the family Chromodorididae that is known to incorporate O-PBDEs, rather than terpenes, in the mantle where the metabolites may play a putative role in chemical defense. The extract of *M. magnifica* was tested in a brine shrimp lethality assay and exhibited an LD_50_ of 58 μg/mL.

## 1. Introduction

Oxy-polybrominated diphenyl ethers (O-PBDEs) are prolific in nature with extensive biological activities. OH- and OMe-PBDEs have been typically reported from sponges of the genus *Lamellodysidea* [1], *Dysidea* [2,3] and *Phyllospongia* [4,5,6] and occasionally from molluscs [7,8,9]. The bioaccumulation of OMe-PBDEs at ppm levels has also been reported in marine mammals, suggesting the persistent nature of these compounds in the environment [10]. The origin of OH- and OMe-PBDEs has been much debated due to their structural similarities to industrial flame retardants [11]. Biosynthetic studies via ^14^C measurements have indicated that OMe-PBDEs [12] as well as OH-PBDEs [13] can be of natural origin. OH-PBDEs have been indicated as the biosynthetic product of sponge-associated cyanobacteria, but the definite genetic basis for algal biosynthesis of OH-PBDEs has not been established [14]. Recent studies, however, have revealed that O-PBDEs are biosynthesized by marine bacteria [15].

Nudibranchs of the family Chromodorididae have been known to sequester defensive terpenes from sponges. As part of our ongoing chemoecological studies, we have reported the terpene chemistry of Chromodorid nudibranchs from various species of *Glossodoris* [16], *Chromodoris* [17], *Goniobranchus* [18] and *Ardeadoris* [19]. To the best of our knowledge, chemical analysis of Chromodorid nudibranchs of the genus *Miamira* has not been reported elsewhere. In this paper, investigation of brominated secondary metabolites of *Miamira magnifica* and *Miamira miamirana* will be described for the first time.

## 2. Results and Discussion

**Figure 1:**
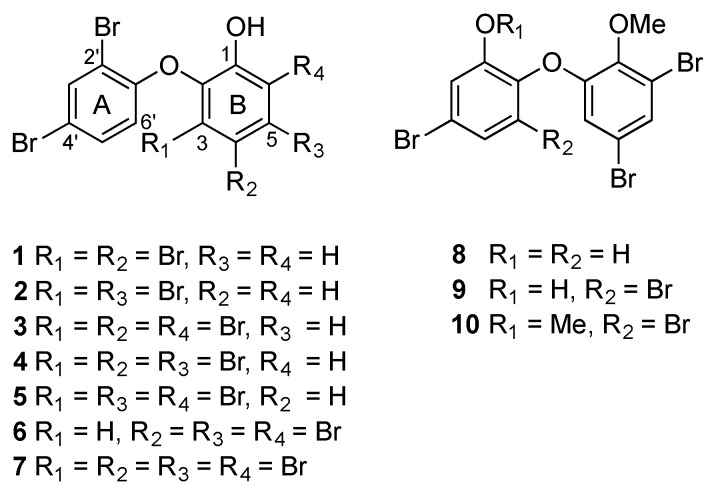
Structure of O-PBDEs **1**–**10**.

A new OH-PBDE **1**, together with known OH-PBDEs **2**–**7** [20,21,22,23], has been isolated from the mantle and viscera extracts of *M. magnifica* (specimen #955). Three known OMe-PBDEs **8**–**10** [4,5,6] have also been obtained from the extracts of *M. miamirana* (#1500) (Figure 1). The diethyl ether extracts of each species were purified by means of normal phase (NP) flash chromatography, followed by reverse phase (RP) or normal phase (NP) High Performance Liquid Chromatography (HPLC). 

Compound **1** was obtained as a colorless oil from NP flash chromatography of the *M. magnifica* mantle extract. A molecular formula of C_12_H_5_^79^Br_4_O_2_ was deduced from the ion peak at *m/z* 496.7028 (calcd. for *m/z* 496.7042) in high resolution electrospray ionization mass spectrometry (HRESIMS) (Figure S1). There were five aromatic protons (δ_H_ 6.40, 6.97, 7.29, 7.47 and 7.79) and a hydroxy signal (δ_H_ 5.70) in the ^1^H nuclear magnetic resonance (NMR) data (Figure S2). The most upfield proton at δ_H_ 6.40 (1H, d, 8.8) was diagnostic for H-6′ in ring A due to the presence of a bromine substituent at C-3 of ring B [24]. In the correlation spectroscopy (COSY) data (Figure S3), the signal for H-6′ was coupled to the signal at δ_H_ 7.29 (1H, dd, 8.8, 2.2, H-5′) that was further *meta*-coupled to the signal at δ_H_ 7.79 (1H, d, 2.2, H-3′). The remaining two *ortho-*coupled aromatic protons at δ_H_ 7.47 (1H, d, 8.8) and δ_H_ 6.97 (1H, d, 8.8) plus two bromine substituents therefore belonged to ring B. 

**Figure 2:**
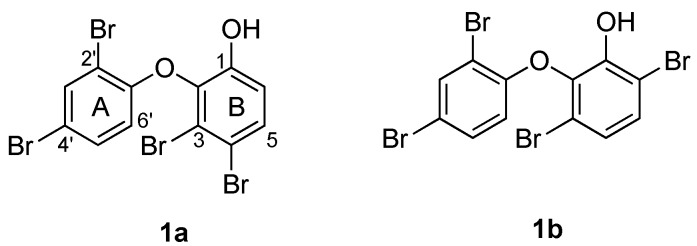
Candidate structures of **1**.

Two plausible structures of **1** with different substitution patterns in ring B were considered (Figure 2). Since one of the two bromine substituents had already been assigned to C-3 based on the chemical shift of H-6′, the other bromine was positioned at either C-4 or C-6. Both proton signals at δ_H_ 7.47 (1H, d, 8.8) and at δ_H_ 6.97 (1H, d, 8.8) showed heteronuclear multiple bond correlation (HMBC) to the carbons at δ_C_ 149.1, 120.0 and 116.0 (Figure S4). Hence, these HMBC data did not differentiate the two candidate structures.

**Figure 3:**
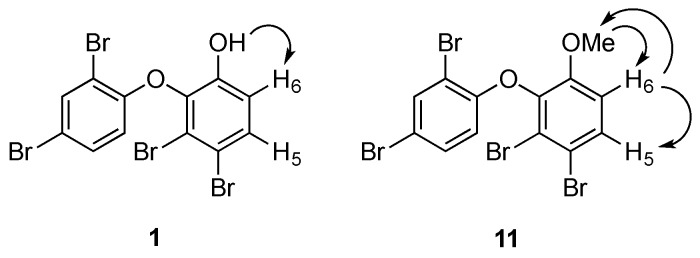
Selected 1D NOE correlations in **1** and **11**.

To solve the data discrepancies, the ^13^C NMR data of ring B in **1** (Figures S4 and S5) were compared to the calculated values [25] for **1a** and **1b** (Table 1). Notably, the experimental ^13^C NMR data matched closely to the expected values for **1a**, suggesting that the metabolite had a 3,4-disubstitution pattern rather than the alternative 3,6-disubstitution pattern. A 1D nuclear Overhauser effect (NOE) experiment was then undertaken to confirm this hypothesis (Figure S6). Irradiation of the hydroxy group at C-1 (δ_H_ 5.70) enhanced the H-6 signal (δ_H_ 6.97), indicating their proximity in the molecule (Figure 3).

For the purpose of confirmation, the phenol group was reacted with methyl iodide to yield a methyl ether analogue (**11**). Two separate 1D NOE experiments were carried out (Figure 3). Irradiation of the methoxy group enhanced the signal of H-6 (Figure S7), while irradiation of H-6 sharpened the signals of H-5 and of the methoxy group (Figure S8). These NOE data confirmed the bromination pattern at C-3 and C-4 in **1**. Thus, compound **1** was proposed as 2-(2′,4′-dibromophenoxy)-3,4-dibromophenol.

The isolated OH-PBDEs **1**–**7** from *M. magnifica* displayed an identical 2,4*-*dibromination pattern in ring A relative to the diphenyl ether bond. Moreover, they also showed identical *ortho-*hydroxylation in ring B relative to the ether linkage with a varying bromination pattern at C-3, C-4, C-5, and C-6. Similarly, OMe-PBDEs **8**–**10** from *M. miamirana* showed an *ortho*-hydroxylation and *ortho*-methoxylation in ring A and/or ring B relative to the ether linkage with an identical 4,6-dibromination pattern in ring B.

The first observation of OH-PBDEs in nudibranchs was reported from *Chromodoris funerea* (*=lineolata*) collected in Iwayama Bay, Palau [7]. Our group reported the isolation of OH- and OMe-PBDEs as well as sesquiterpenes from the digestive tissues of three specimens of the Discodorid nudibranch, *Asteronotus cespitosus*, collected from the Great Barrier Reef and the Phillipines. Since only sesquiterpenes were found in the mantle extracts, it was suggested that *A. cespitosus* selectively incorporated these secondary metabolites from the food source as defensive chemicals. It was also speculated that OH- and OMe-PBDEs were eliminated as they were either too toxic to be integrated into the body tissue, or not as effective as defensive weapons [8]. Unfortunately, the bioactivity of these compounds was not tested.

The Paul group also reported the sequestration of OH-PBDEs in the gastropterid molluscs (Order Cephalaspidea), *Sagaminopteron psychedelicum* and *Sagaminopteron nigropunctatum* that were found feeding on the sponge *Dysidea granulosa* in Guam. The difference between the two molluscs lies in their defense strategies; *S. psychedelicum* has a bright coloration, whereas *S. nigropunctatum* is cryptic. Chemical analysis of the extracts of both species showed the sequestration of **2** as the major component in all body parts. Compound **2** was accumulated in the mantle of *S. psychedelicum* (4.03%) and *S. nigropunctatum* (2.37%) at approximately the same concentration found in the sponge extract. The same metabolite was present at twice the concentration in the parapodia of *S. psychedelicum* (7.97%) and *S. nigropunctatum* (10.10%). In the mucus of *S. psychedelicum*, **2** was detected in trace amounts; whereas in the mucus (1.84%) and egg masses (2.22%) of *S. nigropunctatum*, the level of **2** was quite significant [9].

In contrast to the anatomical distribution of metabolites in *A. cespitosus* [8], OH-PBDEs were found in the mantle and dorsal horn of *M. magnifica* as well as in the digestive tissues (Table 2). Metabolites **1**–**3** were found in all three tissue types, whereas the more highly brominated **4**–**7** were only found in the gut tissues. Compounds **3**–**6** have been reported from the sponge *Lamellodysidea* *herbacea* [22,23]; whereas compounds **2** and **7** were initially isolated from an unidentified Australian marine sponge [20] and the sponge *Dysidea* sp. [21], respectively. The identification of three known O-PBDEs in *M. magnifica* that were also detected from the sponge *L. herbacea*, leads to speculation that *M. magnifica* may sequester the O-PBDEs from this sponge. Likewise, tri- and tetrabrominated OMe-PBDEs **8**–**10** were found in the mantle extracts of *M. miamirana*, as well as in the digestive tissues. Each of compounds **8**–**10** has been reported individually from various genera of sponges but all three compounds have been found in *Phyllospongia* sp. [4,5,6]. It is thus reasonable to propose that *M. miamirana* may feed on this sponge. Our data suggest a preference for sequestration of O-PBDEs with fewer number of bromine substituents into the mantle of these two species.

OH-tetraBDE **2** has been reported to show antifeedant activity against tropical reef fish [26], the gastropod *Stylocheilus longicauda* [27], the pufferfish *Canthigaster solandri* and two species of crab (*Leptodius* spp.) [9,28], highlighting its significant predator deterrent properties. Meanwhile, Handayani and co-workers (1997) reported that the toxicity level of OH-PBDEs against the brine shrimp *Artemia salina* is directly proportional to the number of bromine substituents. Accordingly, OH-hexaBDE **7** displayed the strongest toxicity in the assay with an LC_50_ of less than 1 μg/mL, compared to those of OH-tetraBDEs (LC_50_ 3.30–8.66 μg/mL). Moreover, methylation of the hydroxy group in OH-tetraBDEs significantly reduced the activity in the brine shrimp assay (LC_50_ 26.25 μg/mL) [29]. An extract prepared from two specimens of *M. magnifica* collected near Mooloolaba (#1252-3) was screened and found to exhibit an LD_50_ value of 58 μg/mL against brine shrimp (Figure S9). The extract of *M. miamirana* could not be tested in the same assay due to the limited amount of material. Consequently, direct comparison of the toxicity between the two species could not be drawn. We propose, however, that the sequestration of O-PBDEs with fewer number of bromines in *M. magnifica* and *M. miamirana* may be due to the significant deterrent properties and low toxicity level of the selected O-PBDEs.

To date, *Miamira* is the only known genus of Chromodorid nudibranch that sequester O-PBDEs, rather than terpenes, in the mantle where the metabolites may play a putative role in chemical defense. Some closely-related *Chromodoris* spp. have been shown to selectively accumulate a 16-membered macrolide, latrunculin A, in the mantle parts, leaving other secondary metabolites in the viscera [30]. A recent study has reported the exclusive incorporation of (−)-furodysinin in the dorsal horn of *Ceratosoma trilobatum* and *Ceratosoma gracilimum*, emphasizing the protective function of the dorsal horn in *Ceratosoma* nudibranchs [31]. Compared to *C. funerea* (*=lineolata*) and *A. cespitosus*, *M. magnifica* and *M. miamirana* may also have developed adaptive digestive systems that enable them to consume O-PBDEs without damaging their internal organs. However, further research would need to be undertaken to test this hypothesis.

## 3. Materials and Methods

### 3.1. General Experimental Procedure

NMR spectra were recorded on a Bruker (Karlsruhe, Germany) Avance 500 MHz and 700 MHz spectrometer at ambient probe temperature. All NMR spectra were run in either choloroform-*d* (CDCl_3_) or acetone-*d*_6_ and referenced to solvent signals at 7.26 ppm (^1^H) or 2.05 ppm (^1^H), respectively. The ^13^C NMR data were acquired from the heteronuclear single quantum correlations (HSQC) and heteronuclear multiple bond correlations (HMBC) experiments. Low resolution electrospray ion mass spectra (LRESIMS) were measured using a Bruker Esquire HCT, whereas high resolution electrospray ion mass spectra (HRESIMS) were measured using a Bruker MicroTof Q instrument, each with a standard ESI source. Normal phase flash chromatography was performed using silica gel 60 (40–63 μm; Scharlau, Barcelona, Spain). Normal phase HPLC was carried out using a Waters 515 (Milford, MA, USA) pump connected to a Gilson (Middleton, WI, USA) 132 series refractive index detector with a Waters μPorasil (10 μm, 7.8 × 300 mm) column. Separations were performed using isocratic elution conditions using premixed, filtered and degassed mobile phases. Reverse phase HPLC was conducted on an Agilent (Santa Clara, CA, USA) 1100 series with in-line vacuum degassing unit, an Agilent D1311A quaternary pump, a variable wavelength UV detector, refractive index detector and a Phenomenex (Lane Cove, Australia) Gemini (5 μm, 110 Å, 10 × 250 mm) column.

### 3.2. Biological Material 

A single specimen of *M. magnifica* (specimen #955) was collected from North Stradbroke Island, QLD, Australia, in November 2013. Two individuals of *M. magnifica* (#1252-3) were obtained from Mudjimba, Mooloolaba, QLD, Australia, in March 2014. An individual of *M. miamirana* (#1500) was supplied by Cairns Marine, QLD, Australia, in June 2016. *M. magnifica* (#955) was dissected into mantle, viscera and dorsal horn before chemical analysis. *M. magnifica* (#1252-3) and *M. miamirana* (#1500) were dissected into mantle and viscera only. 

### 3.3. Extraction and Isolation of O-PBDEs 

The mantle, viscera and dorsal horn tissues of *M. magnifica* (#955) were individually extracted with acetone then partitioned against diethyl ether to yield 28 mg of mantle extract, 17.8 mg of viscera extract and 2.3 mg of dorsal horn extract. The mantle extract of *M. magnifica* was subjected to NP flash chromatography using a gradient of hexanes-dichloromethane (DCM)-ethyl acetate (EtOAc)-methanol (MeOH) to yield four fractions. Fraction 1 was further separated by a second NP flash column using a gradient of hexanes-EtOAc to give six subfractions. Subfraction 1b was purified with NP HPLC (5% EtOAc/hexanes) to obtain **2** (0.2 mg) and **3** (0.1 mg). Fraction 3 was also subjected to NP HPLC (5% EtOAc/hexanes) to furnish **1** (0.2 mg), **2** (3.0 mg), **3** (1.5 mg) and **5** (0.1 mg). Fraction 4 was collected as a mixture of **1** and sterol (2 mg). The separation of gut extract of *M. magnifica* was carried out similarly. Fraction 1 from NP flash chromatography was purified by RP HPLC (80% MeCN/water with 0.1% trifluoroacetic acid (TFA)) to afford **2** (2 mg), **3** (0.2 mg), **7** (0.1 mg) as well as fractions containing **3**–**6** (0.5 mg). Compounds **1**–**3** were identified from the dorsal horn extract by data comparison with those of purified compounds. Following the same method, the viscera and mantle tissues of *M. magnifica* (#1252-3) were extracted separately for dereplication analysis. The mantle and viscera extracts of *M. magnifica* (#1252-3) contained the same metabolites as those of *M. magnifica* (#955). 

Extraction of the mantle and viscera tissues of *M. miamirana* (#1500) yielded 6 mg of mantle extract and 3 mg of gut extract. Fraction 3 of NP flash chromatography of the mantle extract furnished a 3:1 mixture of **8** and **9** (1 mg). Fraction 2 also contained **10** as a mixture with sterol (1 mg). Compounds **8**–**10** were also detected in the viscera extract. The IUPAC numbers for O-PBDE congeners isolated in this study is presented in Table S1.

2-(2′,4′-Dibromophenoxy)-3,4-dibromophenol (**1**): colorless oil (0.5 mg); ^1^H NMR (CDCl_3_, 500 MHz) δ 7.79 (1H, d, *J =* 2.2, H-3′), 7.47 (1H, d, *J =* 8.8, H-5), 7.29 (1H, dd, *J* = 8.8, 2.2, H-5′), 6.97 (1H, d, *J =* 8.8, H-6), 6.40 (1H, d, *J* = 8.8, H-6′), 5.70 (1H, s, OH); ^1^H NMR (acetone-*d*_6_, 500 MHz) δ 7.83 (1H, d, *J =* 2.4, H-3′), 7.53 (1H, d, *J =* 8.8, H-5), 7.42 (1H, dd, *J* = 8.8, 2.4, H-5′), 7.08 (1H, d, *J =* 8.8, H-6), 6.52 (1H, d, *J* = 8.8, H-6′), 5.61 (1H, s, OH) (Figure S10); ^13^C NMR (CDCl_3_, 125 MHz) 152.2 (C-1′), 149.1 (C-1), 140.3 (C-2), 136.4 (C3′), 131.6 (C-5′), 131.2 (C-5), 120.0 (C-3), 117.1 (C-6), 116.0 (C-2′, C-4), 115.8 (C-6′), 112.8 (C-4′); HRESIMS *m/z* 496.7028 (calcd. for C_12_H_6_^79^Br_4_O_2_, 496.7042).

### 3.4. Methylation of ***1***

The methylation of **1** was carried out using the method of Dexter et al. (1993) [32] to yield **11.** The ^1^H NMR data of **11** (Figure S11) were consistent with those reported in the literature [33]. 

### 3.5. Brine Shrimp Lethality Assay 

For bioassay purpose, the mantle and viscera extracts of *M. magnifica* (#1252-3) were combined. The brine shrimp lethality assay was conducted based on literature methods [30]. 

## 4. Conclusions

A new OH-PBDE (**1**), along with nine known OH- and OMe-PBDEs (**2**–**10**), has been isolated from the extract of *M. magnifica* and *M. miamirana* collected from North Stradbroke Island, Queensland. Our study suggested that *Miamira* spp. selectively sequestered dietary derived O-PBDEs in the mantle as putative defense metabolites. Our finding highlighted the chemical diversity in Chromodorididae, as it demonstrated the first report of O-PBDEs from this family of nudibranchs. 

## Figures and Tables

**Table 1:** Comparison of experimental and calculated ^13^C NMR data of ring B in **1**.

C	1 ^a^ (exp)	1a ^b^	1b ^b^
1	149.1	147.3	151.6
2	140.3	**148.8** ^c^	**148.8** ^c^
3	120.0	116.6	**112.3** ^c^
4	116.0	118.7	**126.3** ^c^
5	131.2	128.5	128.5
6	117.1	116.6	**109.0** ^c^

^a^ Taken from 2D spectra referenced to CDCl_3_ (δ_C_ 77.16) at 125 MHz; ^b^ Calculated values [25]; ^c^ Bold values indicate ∆δ_C_ > 5.

**Table 2:** Anatomical distribution and percentage weight of O-PBDEs in *M. magnifica* and *M. miamirana*.

O-PBDEs		*M. magnifica*						*M. miamirana*			
		Mantle	% *w*/*w* ^a^	Gut	% *w*/*w* ^a^	Horn	% *w*/*w* ^a,b^	Mantle	% *w*/*w* ^a,b^	Gut	% *w*/*w* ^a,b^
O-triBDE	**8**	-	-	-	-	-	-	X	70%	X	16%
O-tetraBDE	**1**	X	31%	X	6%	X	18%	-	-	-	-
	**2**	X	45%	X	60%	X	64%	-	-	-	-
	**9**	-	-	-	-	-	-	X	25%	X	42%
	**10**	-	-	-	-	-	-	X	5%	X	42%
O-pentaBDE	**3**	X	22%	X	18%	X	18%	-	-	-	-
	**4**	-	-	X	5%	-	-	-	-	-	-
	**5**	trace	2%	X	7%	-	-	-	-	-	-
	**6**	-	-	trace	1%	-	-	-	-	-	-
O-hexaBDE	**7**	-	-	trace	3%	-	-	-	-	-	-

^a^ % weight of compound/total weight of isolated compounds; ^b^ Calculated based on peak integration in the ^1^H NMR spectrum.

## Supplementary Materials

The following are available online at www.mdpi.com/1660-3397/14/11/198/s1, Figure S1: High Resolution ESIMS of **1**, Figure S2: ^1^H NMR (CDCl_3_, 500 MHz) of **1**, Figure S3: COSY spectrum (CDCl_3_, 500 MHz) of **1**, Figure S4: HMBC spectrum (CDCl_3_, 500 MHz) of **1**, Figure S5: HSQC spectrum (CDCl_3_, 500 MHz) of **1**, Figure S6: 1D NOE spectrum (acetone-*d*_6_, 700 MHz) of **1** by irradiating the –OH signal, Figure S7: 1D NOE spectrum (CDCl_3_, 500 MHz) of **11** by irradiating the –OMe signal, Figure S8: 1D NOE spectrum (CDCl_3_, 500 MHz) of **11** by irradiating the H-6 signal, Figure S9: Mortality to brine shrimp (LD_50_) for extract of *M. magnifica* (#1252-3), Figure S10: ^1^H NMR (acetone-*d*_6_, 500 MHz) of **1**, Figure S11: ^1^H NMR (CDCl_3_, 500 MHz) of **11**, Table S1: IUPAC numbers for O-PBDE congeners isolated in this study.
