# Supplementary material for: The Sequestration of Oxy-Polybrominated Diphenyl Ethers in the Nudibranchs Miamira magnifica and Miamira miamirana
Source: Mar Drugs. 2016 Oct 27;14(11):198. doi: 10.3390/md14110198 (PMC5128741; doi:10.3390/md14110198)
Supplement: Supplementary file 1 [file marinedrugs-14-00198-s001.docx]

**Supplementary Materials: The Sequestration of
Oxy-Polybrominated Diphenyl Ethers in the Nudibranchs *Miamira magnifica* and
*Miamira miamirana***

Ariyanti S. Dewi, Karen L. Cheney, Holly H. Urquhart, Joanne T. Blanchfield and Mary J. Garson

# Figure S1. High Resolution ESIMS of 1.


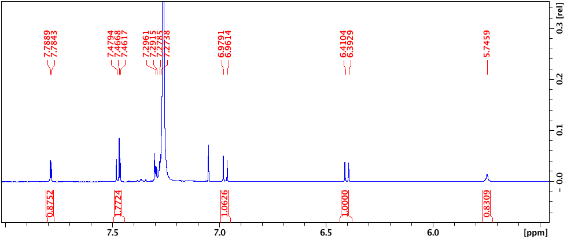


# Figure S2. ^1^H NMR (CDCl_3_, 500 MHz) of 1.


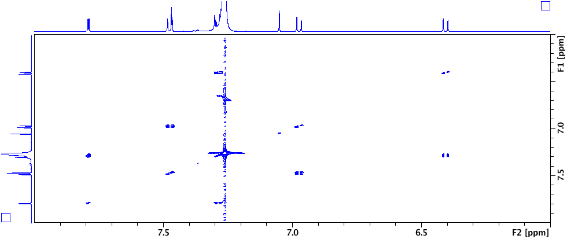


# Figure S3. COSY spectrum (CDCl_3_, 500 MHz) of 1.


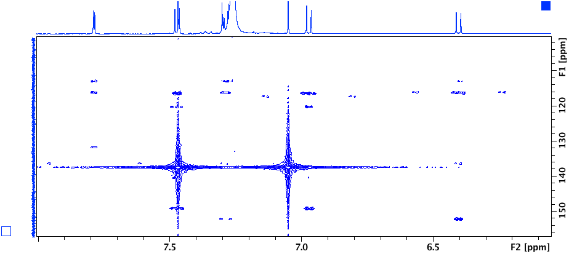


# Figure S4. HMBC spectrum (CDCl_3_, 500 MHz) of 1.


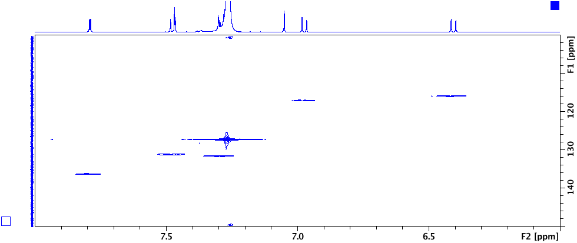


# Figure S5. HSQC spectrum (CDCl_3_, 500 MHz) of 1.

# Figure S6. 1D NOE spectrum (acetone-*d_6_*, 700 MHz) of 1 by irradiating the –OH signal.

# Figure S7. 1D NOE spectrum (CDCl_3_, 500 MHz) of 11 by irradiating the –OMe signal.

# Figure S8. 1D NOE spectrum (CDCl_3_, 500 MHz) of 11 by irradiating the H-6 signal.

# Figure S9. Mortality to brine shrimp (LD_50_) for extract of *M. magnifica* (#1252-3).

# Table S1. IUPAC numbers for O-PBDE congeners isolated in this study.

| **Compound** | **IUPAC Numbers** |
| --- | --- |
| **1** | 6-OH-BDE-42 |
| **2** | 6-OH-BDE-47 |
| **3** | 6-OH-BDE-90 |
| **4** | 6-OH-BDE-85 |
| **5** | 6-OH-BDE-99 |
| **6** | 6-OH-BDE-123 |
| **7** | 6-OH-BDE-137 |
| **8** | 6-OMe-2′-OH-BDE-39 |
| **9** | 6-OMe-2′-OH-BDE-80 |
| **10** | 6,2′-OMe-BDE-80 |


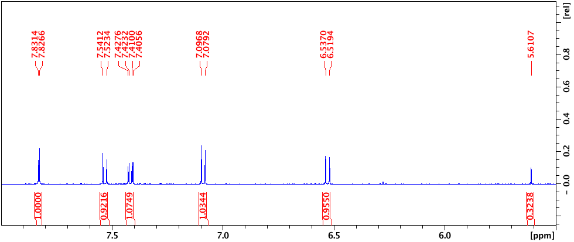


# Figure S10. ^1^H NMR (acetone-*d_6_*, 500 MHz) of 1.


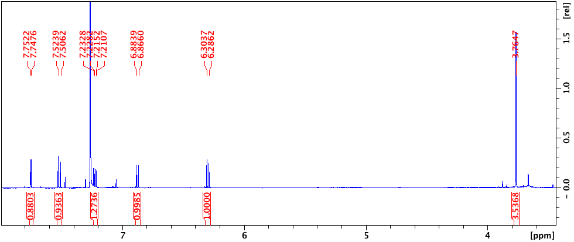


# Figure S11. ^1^H NMR (CDCl_3_, 500 MHz) of 11.
